# Pandemics, privacy, and public health research

**DOI:** 10.17269/s41997-020-00368-5

**Published:** 2020-06-26

**Authors:** Alexander Bernier, Bartha Maria Knoppers

**Affiliations:** Centre of Genomics and Policy, Faculty of Medicine, McGill University, 740, Avenue Dr. Penfield, Suite 5200, Montreal, Quebec H3A 0G1 Canada

**Keywords:** COVID-19, Data sharing, Health policy, Health governance, Pandemic, Privacy, COVID-19, Partage des données, politique de santé, gouvernance de la santé, pandémie, vie privée

## Abstract

Sharing data expediently for pandemic response purposes exposes healthcare providers in Canada to significant regulatory uncertainty. Duplicative and contradictory ethical and legal duties flowing from overlapping sources can stifle flows of medical data among clinicians, researchers, and institutions. Authorities should support caregivers and accelerate research by providing clear guidance to the health sector. Institutions should foster robust data stewardship and standardize their practices to those recognized among the international health informatics community. Reform is critical to ensuring Canadian healthcare providers can deliver efficient health responses that are integrated with dispersed and disparate national and international approaches.

## Résumé

Les chercheurs et cliniciens canadiens désirant intenter le partage rapide des données médicales dans le cadre d’une mobilisation contre la pandémie se retrouvent assujettis à un régime juridique incertain. Les obligations qui leur sont imposées par l’éthique tant que par le droit peuvent s’avérer complexes et contradictoires, en raison du chevauchement de plusieurs politiques, règlements et lois. Il est impératif que les autorités se prononcent afin d’éclaircir les démarches que doivent entreprendre les fournisseurs de soins de santé en partageant les données médicales. Les institutions, quant à elles, devraient concilier leurs pratiques de gouvernance des données à celles employées à l’échelle mondiale par les organismes internationaux œuvrant dans le domaine de la santé. Une solution durable permettant le partage pancanadien et international des données de santé nécessitera désormais la refonte de la législation sibylline actuellement en vigueur.

## Introduction

Worldwide, the COVID-19 pandemic is inspiring clinicians and researchers to pool data and design data repositories for long-term public health research. Ensuring compliance with Canadian privacy regulations creates significant challenges for such efforts. Coordinated and collaborative data stewardship strategies are needed.

## Challenges for collaboration

First, Canada’s privacy laws are non-harmonized for different categories of entities. Private sector, public sector, and health sector entities can find themselves subject to a labyrinthine variety of obligations across their public and private functions. For a pandemic response, this can mean that private-sector clinicians attempting to share data with larger institutional actors such as hospitals and universities will face challenges in ensuring that the privacy law obligations imposed on the clinic and those imposed on the hospital are compatible (Power 2017).

Second, Canada’s private sector privacy laws differ considerably from one province to the next, and the applicable law varies depending on whether data are used within a province’s borders or interprovincially. In the case of internal use, the local provincial laws will apply unless the province has not enacted such laws, in which case Canada’s federal *Personal Information Protection and Electronic Documents Act* (PIPEDA) applies instead. Regarding interprovincial use, PIPEDA will generally apply. In some provinces such as Quebec, provincial and federal authorities disagree as to whether the local provincial law, the federal PIPEDA, or both are applicable to interprovincial data transfers (Power 2017).

Third, certain provinces have strict requirements governing the interprovincial or international use of patient and research participant data. These limits differ from province to province but can operate to restrain clinicians and researchers from hosting or transferring data extra-provincially (Power 2017).

## Challenges for public health and research

For research purposes, Canada’s ethics guidance and laws generally recognize broad consent with appropriate governance (Canada Tri-Council 2018). However, certain provincial laws, such as Alberta’s *Health Information Act* (HIA), impose special requirements on the contents of consent forms that could create difficulties in standardizing pan-Canadian consent materials across the clinical and research settings (Inions et al. 2018). Other provinces impose prohibitive formal requirements on the contents of data transfer agreements and research plans (Dove and Phillips 2015).

Healthcare practitioners face more onerous requirements in obtaining research consent than clinical consent. Research consent must be clearly distinguished from clinical consent (Canada Tri-Council 2018). These requirements could be difficult to discharge systematically due to the high volume of patient inflows experienced during a pandemic, which impose considerable strain on the resources of healthcare institutions.

Canada’s privacy laws create special powers for national public health authorities to requisition and share data liberally across healthcare actors and across provincial borders during a public health emergency without obtaining individual consent (Office of the Privacy Commissioner of Canada [OPC] 2020). However, the permitted purposes of data collection, use, and disclosure in such circumstances are limited to the requirements of emergency response. If clinicians and researchers wish to reuse the data generated for future epidemiological and healthcare research, they may be required to satisfy the more onerous burden of obtaining either individual participant consent or research ethics waivers of consent, coupled with institutional custodians’ approvals, as well as the necessary, project-specific research ethics approvals (Dove and Phillips 2015; Council of Canadian Academies 2015). This requires them to consider the compatibility of the legal requirements applicable to researchers in each sector (private, public, health) and arising in each applicable provincial and federal law (Power 2017). This could force clinicians and researchers to decide between the immediate pooling of data for a robust emergency response, and the slower pooling of data to secure its future availability to researchers.

## Responsive and responsible data stewardship

Faced with ethical and regulatory barriers to efficient data pooling, we impel clinicians and researchers to turn to the governmental actors most capable of orchestrating a coordinated, data-driven response to a health crisis. Ministers and national public health authorities are imbued with exceptional powers to facilitate the transfer and reuse of data for a wide range of purposes. Furthermore, some Canadian privacy laws allow the broad transfer of health data to combat a health risk to known persons, or to provide healthcare to the person that the data concerns (Canada Tri-Council 2018). Privacy Commissioners and other bodies responsible for enforcing privacy laws have expressed willingness to cooperate and facilitate an efficient crisis response (OPC 2020). Healthcare practitioners should consider these options in opening immediate channels for data sharing.

For a coordinated long-term response to epidemic threats, including COVID-19, Canadian researchers should coordinate their data collection and data governance practices with those of established international consortia to facilitate efficient data deposit (Council of Canadian Academies 2015). Institutional research ethics committees, in coordination with institution heads, can agree to a singular multi-site ethics review of an international or international research project to increase efficiency and ensure the consistency of collection and storage practices across research sites (Canada Tri-Council 2018; Council of Canadian Academies 2015). Institutions can manage their data releases using Data Access Committees (DACs) and Data Access Compliance Offices (DACOs), teams of multidisciplinary scientific members and ethico-legal specialists, to ensure the compliance of each data release to ethics, law, and local policies (Joly et al. 2012). Data can be made anonymous using statistical or algorithmic deidentification methods or appropriate aggregation methods and circulated openly, but anonymized data often retain only negligible scientific utility (Langarizadeh et al. 2018).

The COVID-19 pandemic has lain bare the fault lines in Canada’s health data regulation. Fundamental reforms will be necessary to enable efficient responses to future health emergencies and to ensure that Canadian healthcare providers are not unduly precluded from granting Canadians high-quality medical care as data-driven approaches continue to reshape the health sector (Beauvais and Knoppers 2020).

## Proposed regulatory reforms

The use of localized, divergent rules to govern data processing activities in Canada and other jurisdictions, including the European Union, has attenuated international research collaboration for fear of legal non-compliance (Peloquin et al. 2020). Health data custodians in Canada have favoured the creation of data repositories that are provincial or purpose-specific rather than multipurpose or pan-Canadian, due to the complexities inherent in ensuring holistic privacy law compliance (Council of Canadian Academies 2015).

Healthcare entities would benefit from the streamlining of regulatory obligations across sectoral and territorial boundaries. Requirements to restrict data reuse to the initial purposes of collection, or to retain data within prescribed territorial boundaries, are difficult to reconcile with the international nature of a collaborative pandemic response, and the potential to use data for flexible secondary purposes (Council of Canadian Academies 2015).

Government and industry stakeholders, as well as academic commentators, have recognized that procedural mechanisms for regulating privacy are difficult to reconcile with continuous technological disruption and innovation in the digital age; technology-neutral, principle-driven protections are more adaptable to the ever-shifting context of data use than are formalistic rules (Department of Justice 2019). Canada’s Department of Industry, Canada’s Department of Justice, and the OPC have made recent proposals for reform. The establishment of data trusts, banks of data that are created for broad reuse for approved purposes, is proposed. Also propounded are exceptions to the requirement to obtain consent to collect, use, or disclose data for purposes in the public interest or arising in the ordinary course of business. Streamlining enforcement procedures across provincial and sectoral boundaries is also suggested. Increased audit and enforcement powers for regulators are endorsed, and heightened individual rights to control, transfer, and erase data are advocated for individuals. In sum, the recommendations favour decreased procedural burdens for data use generally, counterbalanced by increased substantive protections for individuals against unconscionable uses of their data (Department of Justice 2019; Innovation, Science, and Economic Development Canada 2019; OPC 2019). We propose regulatory approaches to actualize these policy objectives.

## Harmonizing data stewardship and regulatory oversight

First, the regulatory regime applicable to health research consortia should be harmonized across national and sectoral boundaries, whether by legislation or through the standardization of enforcement practices by Privacy Commissioners (OPC 2019). Uniform privacy laws across Canadian provinces can foster increased predictability and public trust, as privacy obligations will be consistent across all public institutions. Canadians are thus assured that their data are subject to the same protections irrespective of the institution responsible for their safekeeping. Furthermore, the synchronization of privacy regimes allows for the central administration of health data. Central data management prevents the replication of collection and storage processes for the same data across multiple repositories. To implement such a method without derogating from the shared federal and provincial oversight of health data, privacy regulators can subject government-created data repositories to delegated oversight performed by a designated Privacy Commissioner in accordance with a commonly chosen privacy law, irrespective of the provenance of the data stored in the repository. In research ethics oversight, such imperatives have prompted the adoption of delegated review models, wherein one research ethics board performs ethics review of a research project across multiple sites (Zawati et al. 2015). An equitable pandemic response will benefit from consistent regulation and coordinated oversight.

Second, blanket doctrines of purpose limitation and temporal limits to the use and storage of data should be disfavoured. Instead, individuals should be granted fundamental rights in their data, drawing from human rights legislation such as the *Charter of Rights and Freedoms*. Such rights must be supported by broad audit and enforcement powers apportioned to Privacy Commissioners, directly actionable rights in data for patients and research participants, and obligations for data custodians to demonstrate that privacy has been accounted for in organizational structure and technological design (OPC 2019). In empowering individuals and Privacy Commissioners to hold entities accountable for their uses of health data, legislators can ensure that the interests of vulnerable groups are not disregarded, as they might be in the present model that relies principally on self-regulation. A rights framing enables the explicit balancing of individual, community, and public interests in data. Justly balancing such interests during a pandemic is best achieved if consideration thereof is enshrined in the law.

Furthermore, health data are a limited resource that must be used responsibly, and not destroyed callously. Whereas it is sensible to establish maximum retention periods for personal data collected in transactional contexts, health data represent a finite resource, the collection of which can often subject individuals to hardship and sacrifice (Canada Tri-Council 2018). Using destruction obligations and preclusions on data reuse to safeguard health data can further stigmatize marginalized populations, and deny vulnerable individuals access to research benefits (Canada Tri-Council 2018). Leveraging longitudinal data from past epidemics can reduce the need for primary data collection, protecting individuals from potential health risks incurred in research participation.

Third, data localization requirements mandating data to be retained in its territory of provenance, and formal obligations that consent or compliance documentation be structured using a predetermined format, should be replaced with a more holistic obligation to ensure that data are held to an equivalent standard of protection across jurisdictions, as some Canadian provinces already do (Inions et al. 2018; Power 2017). This encourages health data custodians to hold downstream recipients of data accountable using contractual agreements and data audit provisions tailored to the risks inherent in the specific data uses anticipated (e.g., cloud storage, algorithmic processing). Furthermore, it prevents inconsistencies in formal compliance obligations across jurisdictions from precluding otherwise responsible data transfers. Mobilizing accurate, voluminous, and compatibly formatted data across borders is a prerequisite to a public health response that ensures equitable access to scientific knowledge for all communities.

## Lessons for the future

Challenges faced in sharing health data to respond to the COVID-19 epidemic demonstrate how Canada’s duplicative privacy legislation can create considerable barriers for health data sharing in Canada with minimal gains in personal privacy. The failure to standardize Canada’s health data regime imposes considerable institutional costs and legal risks on the healthcare sector and causes clinicians and researchers to eschew data centralization and secondary data use (El Emam et al. 2011). Reform is the only viable solution for the future, and the future is now!
